# Scenario modelling as planning evidence to improve access to emergency obstetric care in eastern Indonesia

**DOI:** 10.1371/journal.pone.0251869

**Published:** 2021-06-09

**Authors:** Frederika Rambu Ngana, A. A. I. N. Eka Karyawati

**Affiliations:** 1 The Northern Institute, Charles Darwin University, Darwin, Northern Territory, Australia; 2 Faculty of Science and Technology, Nusa Cendana University, Kupang, Nusa Tenggara Timur, Indonesia; 3 Faculty of Mathematics and Natural Sciences, Udayana University, Denpasar, Bali, Indonesia; Al Mansour University College-Baghdad-Iraq, IRAQ

## Abstract

The rate of maternal deaths in remote areas in eastern Indonesia–where geographic conditions are difficult and the standard of infrastructure is poor–is high. Long travel times needed to reach emergency obstetric care (EMOC) is one cause of maternal death. District governments in eastern Indonesia need effective planning to improve access to EMOC. The aim of this study was to develop a scenario modelling tool to be used in planning to improve access to EMOC in eastern Indonesia. The scenario model was developed using the geographic information system tool in NetLogo. This model has two inputs: the location of the EMOC facility (*PONED*) and the travel cost of moving across geographical features in the rainy and dry seasons. We added a cost-benefit analysis to the model: cost is the budget for building the infrastructure; benefit is the number of people who can travel to the EMOC in less than 1 hour if the planned infrastructure is built. We introduced the tool to representative midwives from all districts of Nusa Tenggara Timur province and to staff of Kupang district planning agency. We found that the tool can model accessibility to EMOC based on weather conditions; compare alternative infrastructure planning scenarios based on cost-benefit analysis; enable users to identify and mark poor infrastructure; and model travel across the ocean. Lay people can easily use the tool through interactive scenario modelling: midwives can use it for evidence to support planning proposals to improve access to EMOC in their district; district planning agencies can use it to choose the best plan to improve access to EMOC. Scenario modelling has potential for use in evidence-based planning to improve access to EMOC in low-income and lower-middle-income countries with poor infrastructure, difficult geography conditions, limited budgets and lack of trained personnel.

## Introduction

Maternal mortality is a major health challenge in most low-income and lower-middle-income countries today [1]. Travel time is critical for patients seeking emergency maternal health care. The high number of maternal deaths is caused in part by geographical inaccessibility to emergency obstetric care (EMOC) [2]. The difficult geographic conditions result in long travel times to reach EMOC [3]. This situation is worse in the wet season when some areas are flooded [3, 4].

Indonesia has high maternal mortality. In 2019, the maternal mortality ratio (MMR) in Indonesia was 305 per 100 000 live births [5]. In 2005, the Ministry of Health of the Republic of Indonesia established PONED (*Pelayanan Obstetri Neonatal Emergensi Dasar*), a national program to provide emergency obstetric care and decrease MMR at district level [6–8]. Although MMR has improved, the number of maternal deaths is still high, especially in remote locations. The causes of maternal death are well known. Slow travel in the wet season to receive EMOC is one cause of maternal death [3]. For example, in the most remote areas of eastern Indonesia, travel times to reach obstetric care are more than 8 hours [9]. According to the 2013 PONED guidelines [10], each PONED should be located less than 1 hour by public transport from the village and from standard (non-PONED) clinics. Travel times from a PONED clinic to hospital should be less than 2 hours. The PONED should service catchment areas of 50 000–100 000 individuals [10]. The Indonesian Government needs to consider travel time in planning to improve access to EMOC and use the Indonesian Ministry of Health guidelines [10] to make planning decisions to improve access to emergency obstetric care at district level.

In this study, we developed an interactive scenario modelling tool that, combined with a cost-benefit analysis, may be used for infrastructure planning to improve access to services. As a case study, we applied our tool for improving access to EMOC in the Kupang district, eastern Indonesia.

We developed the scenario modelling tool using the geographic information system (GIS) in NetLogo, a free programming language and integrated development environment for agent-based modelling [11]. The scenario modelling includes a cost-benefit analysis (CBA) that can be used to choose between alternative scenarios [12]. CBA is used in city planning when budget constraints apply [13]. Lichfield [13] suggested using a ‘with and without’ approach in applying CBA to city planning. Planning proposals can be compared using this approach–for example, by evaluating what would happen with and without the planning. This is a dynamic approach. In our tool, CBA assesses the cost of infrastructure against benefit, measured as the number of people who can reach EMOC in less than 1 hour. We modelled time to reach EMOC and calculated the number of people who travel to EMOCs in each of several time periods.

We introduced the scenario modelling tool to district planning agencies and midwives in two separate focus group discussions to collect information on the perceptions of participants on the usefulness of the modelling tool to improve access to services. We video-recorded the discussions and then analysed the recordings using version 11 of the qualitative data analysis computer software package NVIVO.

## Literature review

### Modelling access to emergency obstetric care

Access to health services is influenced by economic conditions, topography and infrastructure. Modelling travel time is an effective way to measure ease of access to services such as EMOC in regions of low income, poor infrastructure, and difficult geographic terrain. For example, Bailey et al. [14] measured time taken to transfer patients needing emergency obstetric and newborn care from first-level facilities to referral facilities in the Tigray and Amhara regions in Ethiopia, a country which has sparsely populated mountain areas where road infrastructure is poor [2]. Chen et al. [15] modelled travel time to reach emergency obstetric and neonatal care in the Kigoma Region, Tanzania, where geographic access to such care is poor. Makanga et al. [4] modelled travel time to maternal health services in southern Mozambique, which has poor infrastructure; almost 90% of the roads are unpaved, of which 75% are minor unplanned roads. In the wet season, roads are difficult to traverse; when flooded, they are unusable. Myers et al. [16] modelled travel time to EMOC in Timor Tengah Selatan (TTS) district, eastern Indonesia. The terrain of the district is mainly rough, there are few roads, and those are in poor condition. In the rainy season, the roads are difficult to traverse because of flooding or landslides.

Travel was modelled using two inputs: travel time and cost. The first was based on the location of health services and the second on the cost of movement across each geographical feature [17]. Geographical features were entered as land cover raster grids that combined vegetation, transport infrastructure (roads), watercourses and elevation data [18]. Each of the land cover grids assumed different speeds of travel. For example, Okwaraji et al. [17], when modelling time to travel to health facilities in Ethiopia, assigned a walking speed of 5 km/h across level ground, and 0.1 km/h up slopes greater than 30 degrees and through water. Myers et al. [16], when modelling time of travel to EMOC in a district in eastern Indonesia, used local knowledge to define travel speed, based on type of transportation: by ambulance (car), public transport (bus), and walking through different types of land cover and weather conditions (dry and wet seasons).

In this study, travel time was generated with GIS software using raster analysis techniques. One example of raster analysis software used for modelling travel time is AccessMod [1, 4, 16, 19–21], developed by the World Health Organization (WHO), which uses cost-distance analysis to model accessibility of health services. Initially a plugin in ArcView, AccessMod is currently an extension in ArcGIS 9.3.1. Other studies have used cost analysis on IDRISI Taiga GIS software to calculate travel time to health posts [22].

However, GIS software generates only a static model of travel time, while travel time is dynamic. Myers et al. [16] stated that simplistic scenarios cannot capture the variability of changing weather; they suggested using an interactive model to capture various scenarios. Therefore, to capture a thousand various travel time scenarios, Fisher and Lassa [18] developed a geo-simulation interactive tool using GIS format in NetLogo [11]. Travel time is calculated automatically through geo-simulation. We followed the method Fisher and Lassa [18] but added a cost-benefit analysis.

### Geo-simulation

Modelling travel time can be simulated using cost-distance transform and chamfer metrics [18]. This algorithm calculates distance between neighbouring cells on a grid [23]. Fisher and Lassa [19] calculated distance from the centre point using the 5 × 5 distance transform mask. Calculation of cell distance is done by scanning vertically from top left to bottom right corner, then bottom right to top left corner, then repeated as a horizontal scan. The process scans a square or rectangular lattice by placing a mask over a cell in the grid. The lattice can be a single source or destination point, or a set of points which are automatically generated. The destination points are initialised to 0 and all other points are assigned as large values, for example, 9999 [23] or 99 999 [18]. Points assigned large values were designated barriers or inaccessible areas. The scan calculated travel time at each point from the central cell to neighbouring cells, using speed values of each cell multiplied by its fractional value, 0.9866, 1.4141 or 2.2062 [18, 23]. The minimum calculated travel times of surrounding cells are added to the current cell travel time value if they are smaller than the current value. After scanning, the distance to the nearest point in the set of source points is assigned to each cell in the final lattice.

## Methods

### Study area

This study was undertaken in the Kupang district in the province of Nusa Tenggara Timur (NTT) in eastern Indonesia. The district was selected as a case study having high maternal deaths. The regional medium-term development plan of Kupang district planned to decrease the MMR to 225 per 100 000 live births by 2015 [24]. However, the district did not achieve this target: in 2018, it was 257 [25]. Most maternal deaths occurred at home because of difficulty of access to the closest clinic [24].

The population of Kupang district in 2019 was 380 900 people across three islands (Timor, Semau and Kera) [26]. Most people live on Timor and Semau islands. Only 82 families live on Kera island [27].

**Fig 1:**
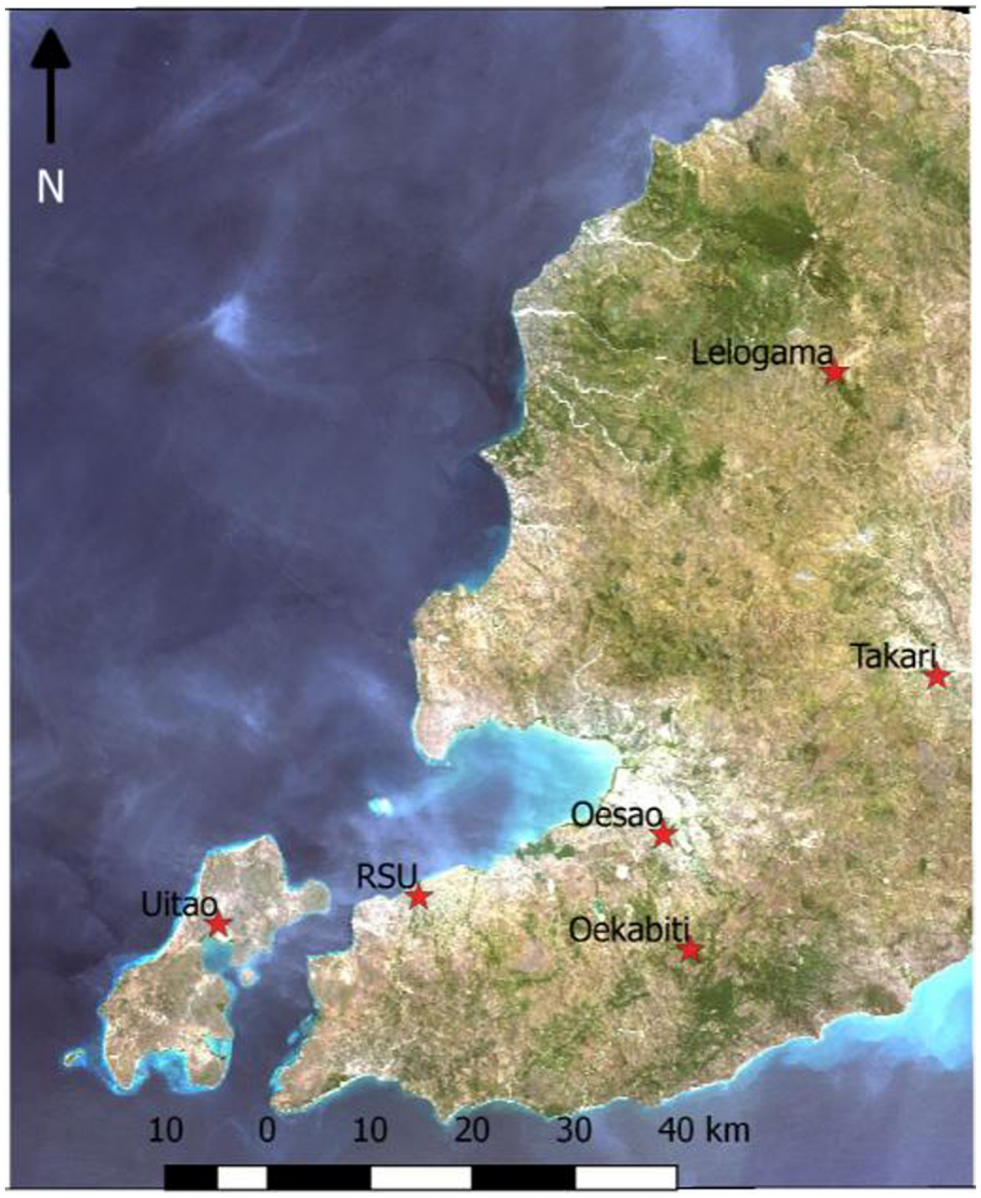
Locations of PONED and PONEK in Kupang district in the red stars.

The district has five EMOCs: Puskesmas Oesao, Oekabiti, Takari, Uitao (on Semau Island) and Lelogama. There is one PONEK (*rujukan obstetri dan neonatal emergensi/komplikasi*) at a public hospital, RSU (*Rumah Sakit Umum*), which provides comprehensive emergency obstetric care (Fig 1).

### Data collection

When developing the scenario modelling tool, we collected datasets as input for the model and software from 8 sources:

1. Five geographical coordinates of the location of PONEDs (EMOC) and PONEKs were taken using a handheld global positioning system (GPS) device, Garmin eTrex^®^ 30x.
2. Satellite imagery from Landsat 8 (Path: 111/67) [28] and DEM (digital elevation model) of Timor Island taken in 2016 [29] were used.
3. Datasets of road networks in 2016 were obtained from *bappeda* (district planning agencies). ‘Missing’ roads, that is, those not on the maps, were digitised using satellite imagery in QGIS [30]. The classes of roads were (1) national, (2) provincial, (3) district, (4) village.
4. Roads to the locations of PONEDs and planning meetings were plotted from Oelamasi, the capital city of Kupang district.
5. The residential map from *bappeda* in JPEG format was digitised into SHP format by the first author [30].
6. Maps of administration boundaries were obtained from *bappeda*.
7. GPS datapoints of the location of rivers and broken roads were collected.
8. GPS ground references of each land cover type (forest, savanna, ocean, farm and residential, river) were taken.

We used three free simple GIS software packages to create the travel time scenario model:

1. Saga [31]
2. QGIS: Quantum GIS [30].
3. NetLogo [11]

### Development process

**Fig 2:**
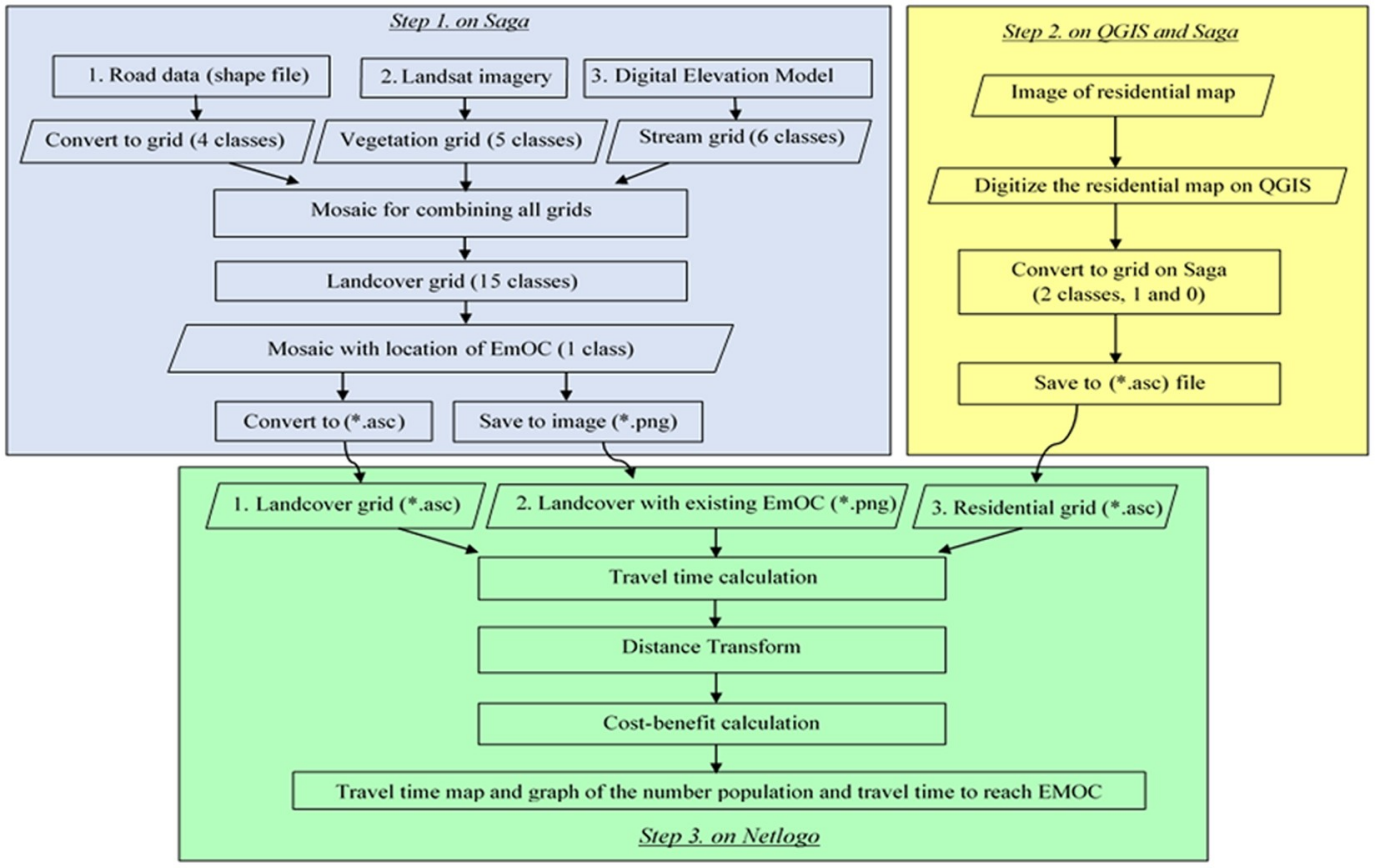
Design of scenario modelling tool.

This section presents the development process of the scenario modelling (with cost-benefit analysis) tool for infrastructure planning. We followed three steps (Fig 2).

**Fig 3:**
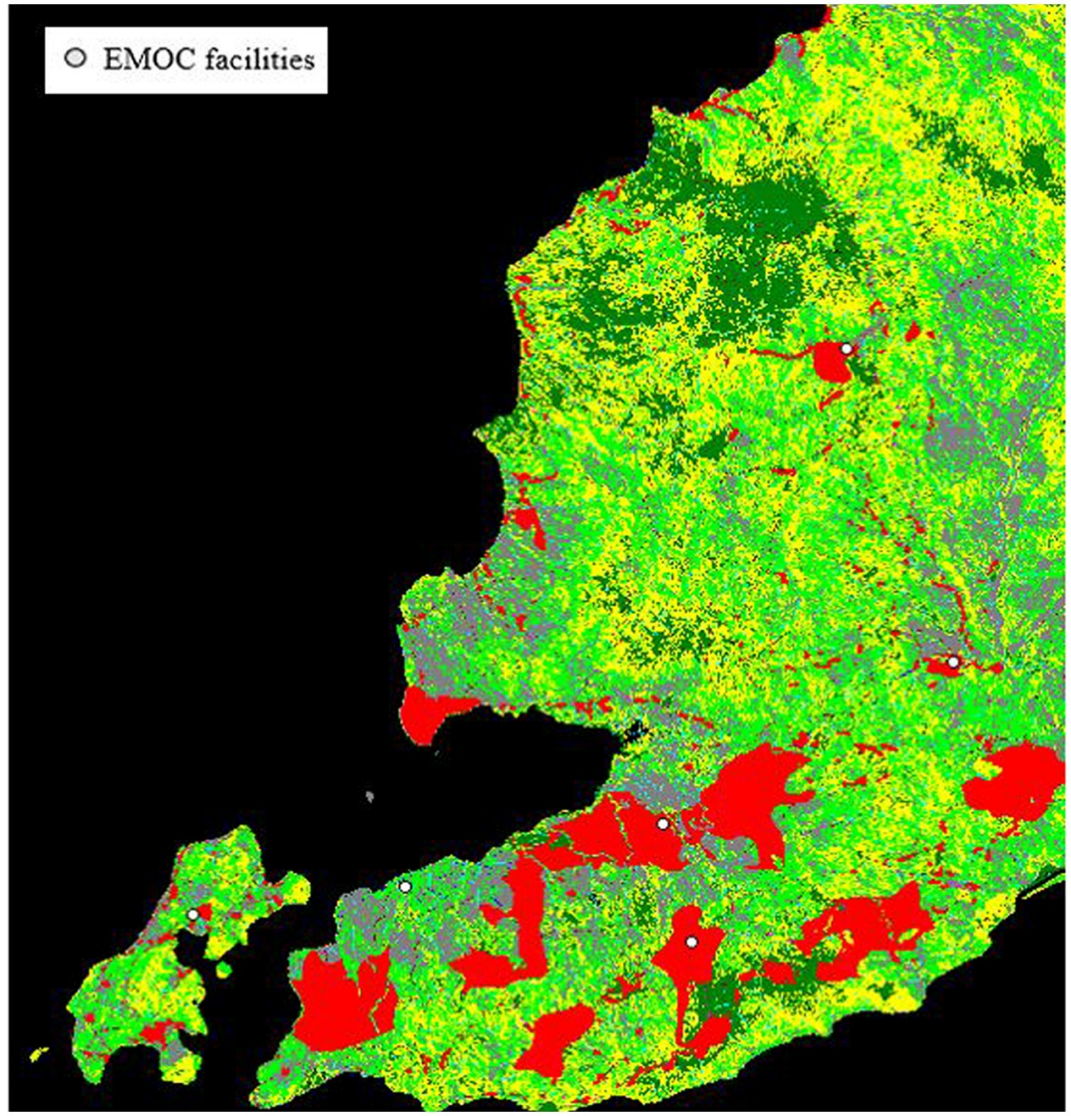
Grid layer of residential areas in red. The white dots as EMOC facilities.

**Step 1**: Creating land cover grids on Saga. This required input from three datasets: roads, Landsat imagery and DEM.
  All data layers were projected onto the spatial reference frame WGS84/UTM 51S, then cropped to the administrative boundaries of each EMOC catchment area. The Landsat image was reclassified, using an unsupervised classification, to derive vegetation grid layers (forest, savanna, ocean, farm and residential). The DEM was used to produce a watercourse grid (stream classes 1–6) by implementing a Fill Sinks operation producing a rivers network vector with Strahler order [18]. The roads data was classified into four types: village, district, provincial, and national. The roads layer and rivers network layer were gridded into the raster grid layer. The EMOC point locations were gridded as EMOC layers. We assigned the ID value of 1000 for EMOC. Then the four raster layers (vegetation, roads, rivers network and EMOC) were mosaicked into one land cover grid layer consisting of 16 classes. Each class has a different travel time, calculated in seconds for each 30-metre cell (1):
$$TT=30/(((\frac{Travel\;speed}{3600})))$$ (1)
  TT: Travel Time in seconds; *Travel speed* in km/hour. Results for each class are shown in Table 1.
**Step 2**: The district residential image in JPEG format was digitised in QGIS to shapefile format, then gridded into a raster layer in Saga (Fig 3) with two classes, 1 and 0 (0 for *no data*).
**Step 3**: The grid layers of land cover from Step 1 and the residential grid layers from Step 2 were then converted into the ASC format used for creating the dynamic travel time scenario modelling tool on NetLogo. The PNG image of the land cover grid, which shows the location of EMOCs, was used as background of the interface of the tool. The two ASC files of land cover and residential grids were used to calculate travel time. Travel speed across each class of land cover was assigned on the NetLogo interface. The location of any new EMOC was defined by users.

**Table 1:** Travel time and speed for each class of landcover.

	ID	Cover Class	Travel Speed	Travel Time
			(km/h)	(seconds)
1	1	Forest	1	180
2	2	Savanna	2	90
3	3	Ocean	0	99 999
4	4	Farm	0.75	240
5	5	Residential	3	60
6	101	Stream Class 1	2	90
7	102	Stream Class 2	2	90
8	103	Stream Class 3	2	90
9	104	Stream Class 4	0	99 999
10	105	Stream Class 5	0	99 999
11	106	Stream Class 6	0	99 999
12	201	Village road	5	24
13	202	District road	15	12
14	203	Provincial road	30	6
15	204	National road	60	3
16	1000	EMOC	0	0

**Fig 4:**
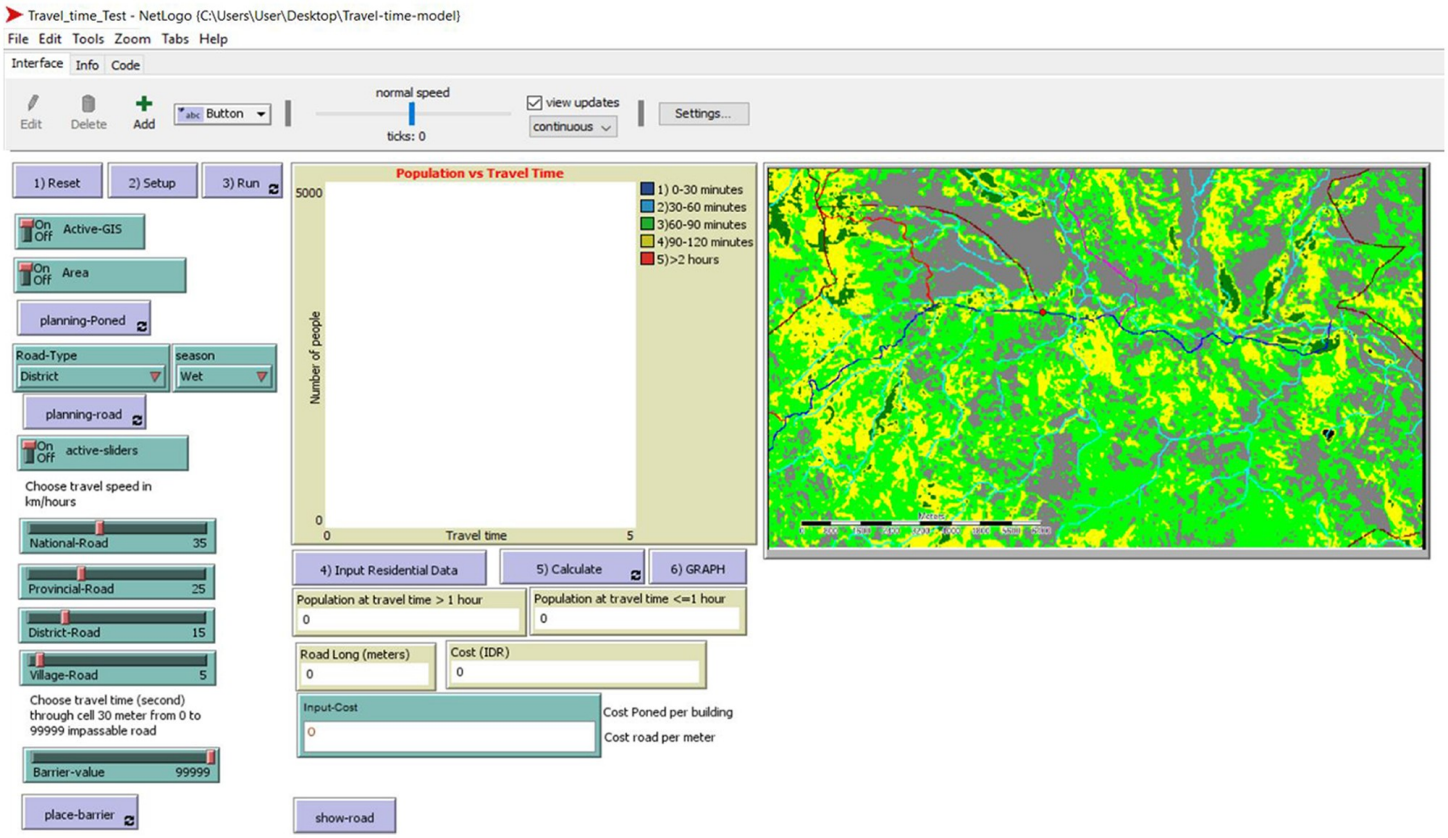
Interface of the travel time scenario modelling tool.

To create the scenario modelling tool, we set up the interface in NetLogo based on the model design (Fig 4).

The interface consists of 16 items; four coding operations were performed for each of these items.

**Fig 5:**
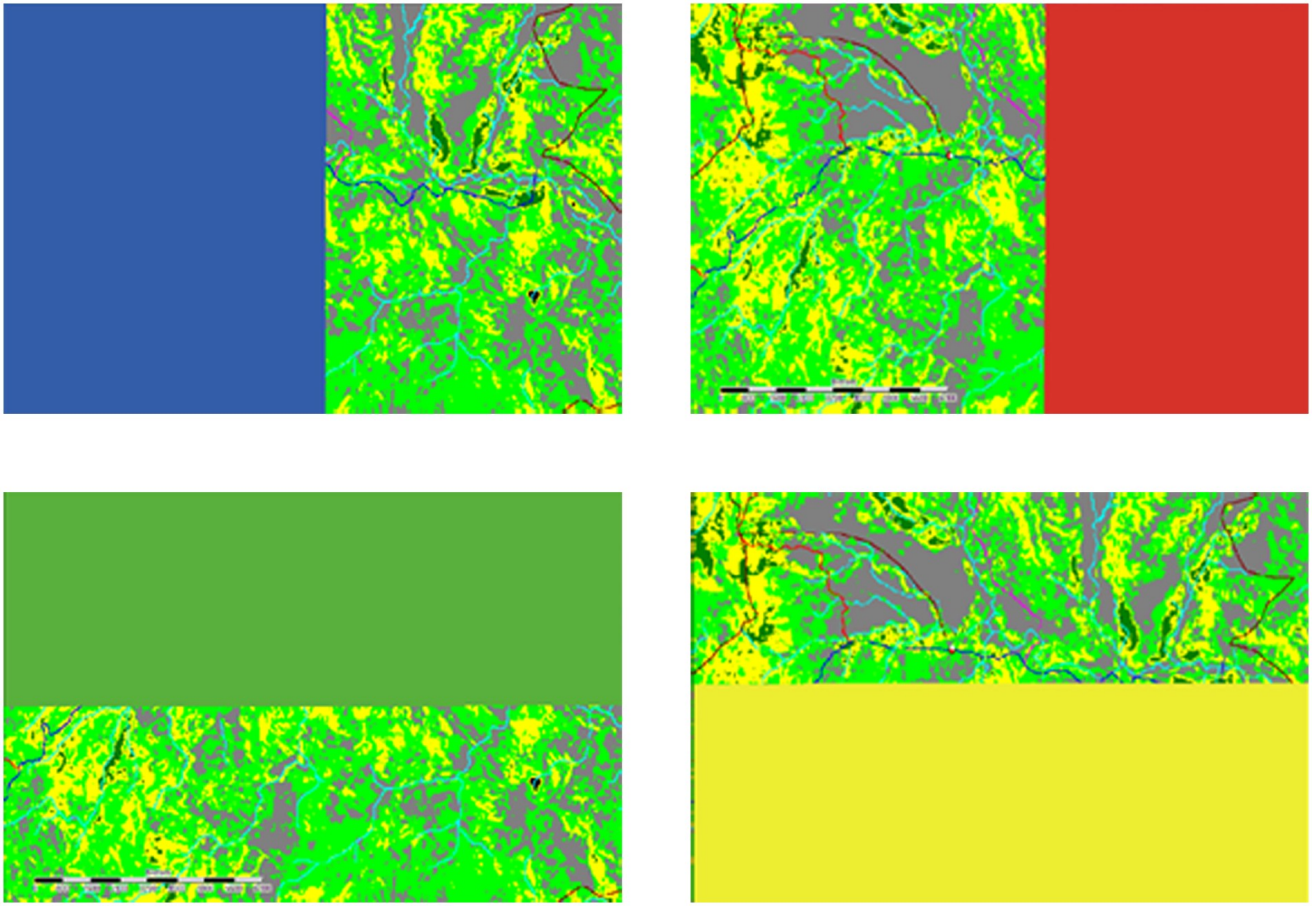
Scanning process. Clockwise from top left: forward scan, back scan, top scan and bottom scan.

**Fig 6:**
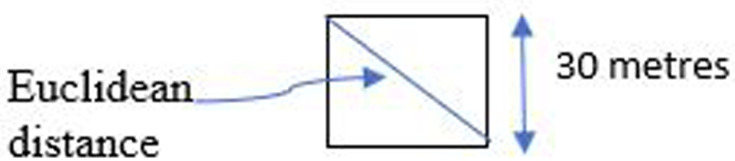
Pixel size of each patch.

1. **Reset procedure**. This procedure clears all data and sets the patch colour based on travel time ranges.
  if (travel-dist / 60) > = 0 and (travel-dist / 60) < = 30 [set pcolor 103];blue
  if (travel-dist / 60) > 30 and (travel-dist / 60) < = 60 [set pcolor 94]; sky
  if (travel-dist / 60) > 60 and (travel-dist / 60) < = 90[set pcolor 55]; green
  if (travel-dist / 60) > 90 and (travel-dist / 60) < = 120 [set pcolor 45]; yellow
  if (travel-dist / 60) > 120 and (travel-dist / 60) < = 9999 [set pcolor 14]; red
  if (travel-dist / 60) > 9999 and (travel-dist / 60) < = 9999999 [set pcolor 0]; black
2. **Setup procedure**. This procedure sets up the screen with GIS data uploaded from the ASC land cover grid file.
  ask patches [set cost 0 set dist 999999]; set cost 0 for distance 999999 from PONED
  ;;load landcover layer
  set covergis gis:load-dataset "data/LC-Takari.asc"
  resize-world 0 gis:width-of covergis 0 gis:height-of covergis
  gis:set-world-envelope
  (gis:envelope-of covergis)
  if active-gis [gis:apply-raster covergis cover set-cost]
  if active-gis [import-pcolors-rgb "data/Takari.png"]; input image map
  ask patches with [cover = 1000] [sprout-dest 1 [set shape "cylinder" set size 2 set color white]]; PONED was assigned as 1000
  ask patches with [cover = 1000] [set dist 0]
3. **Simulation procedure**. This procedure implemented the distance transform (DT) and chamfer scan [23] to calculate the minimum distance between each point cell. As an example, the DT formula for forward scan at patch (-1 2) is written as:
  Ifelse (([cover] of patch-at -1 2 = 1) or ([cover] of patch-at -1 2 = 2) or ([cover] of patch-at -1 2 = 3) or ([cover] of patch-at -1 2 = 4) or ([cover] of patch-at -1 2 = 5) or ([cover] of patch-at -1 2 = 101)or ([cover] of patch-at -1 2 = 102)or ([cover] of patch-at -1 2 = 103)or ([cover] of patch-at -1 2 = 104)or ([cover] of patch-at -1 2 = 105)or ([cover] of patch-at -1 2 = 106) or ([cover] of patch-at -1 2 = 201)or ([cover] of patch-at -1 2 = 202) or ([cover] of patch-at -1 2 = 203) or ([cover] of patch-at -1 2 = 204)) [set LDME1 (C1 * cost + ([dist] of patch-at -1 2))] [set LDME1 999999]
  where LDME1 is the least distance mask element 1. The fractional values for the DT are:
  set a1 0.9866 set b1 1.4141 set c1 2.2062
  Four scanning processes were applied: forward scan, back scan, top scan and bottom scan (Fig 5).
  Before scanning, the travel cost of each cell grid was set, based on land cover type (previously assigned on Saga). Travel cost of roads and Stream 3 were defined in NetLogo. Travel cost of roads was taken from the value of each road’s sliders in the Netlogo interface; these were used to assign the travel speed (in km/h) on national, provincial, district and village roads. Planned new roads were created by drawing them in using the code shown below:
  if (mouse-down?)
   [ask patch mouse-xcor mouse-ycor
    [
    set pcolor 30; change this color coding for boat’s route to white
    ;set pcolor white
    ifelse Active-GIS [
    if (Road-Type = "National") [set cover 204]
    if (Road-Type = "Provincial") [set cover 203]
    if (Road-Type = "District") [set cover 202]
    if (Road-Type = "Village") [set cover 201]]
    [set cost 2]
      ]
   ]
  Travel cost of Stream 3 varied with the weather. It is a big river that can be crossed in the dry season, but it floods in the rainy season and is impassable. Therefore, the dry season travel cost was set at 108, but at 99 9999 for the rainy season (i.e. stream number 3 is a barrier). A PONED location can be drawn by clicking the mouse, causing a white circle to appear on the map. The code is:
  if (mouse-down?)
   [ask patch mouse-xcor mouse-ycor [sprout-bar 1
    [set cover 106 ask patches in-cone 1 360 [set pcolor black] die]
    ] ]
  The barrier was drawn by giving the patch the same value as that of land cover number 106, which has cost 99 999. The code is:
  if (mouse-down?)
  [ask patch mouse-xcor mouse-ycor [sprout-dest 1
  [set dist 0 ask patches in-cone 2 360 [set pcolor white] die]]]
4. **Cost-benefit procedure**. Cost-benefit analysis was undertaken using cost of infrastructure and benefit of people who can travel to EMOC in less than 1 hour. Cost of road building was calculated by multiplying the road length with the price of road each metre. To get the road length, we first calculated the Euclidean distance for 30-metre pixels (Fig 6), the cell size of the patch, with Eq (2).
$$road\;length=number\;of\;patches\;\times \;\sqrt{{\left(\frac{30}{2}\right)}^{2}+{\left(\frac{30}{2}\right)}^{2}}$$ (2)

The cost of road building can be calculated by Eq (3):

cost=inputcost*countroad*sqrt((15*15)+(15*15)) (3)

Where inputcost is the cost of each metre of road and countroad is the total road length.

In the cost-benefit analysis, the benefit is the number of people able to travel to EMOC for each travel time zone. It was calculated using population density for each EMOC catchment area and residential grid cell. Each grid cell on the map has 30 m resolution per pixel. If the population density is 68 people/km^2^ for each grid cell, the number of people per cell is equal to (30 x 30 x 68/1 000 000).

The residential grid cells map was gridded from a residential location map of Kupang district on Saga (Fig 6). The map was then converted into ASC format, uploaded into NetLogo, and the number of people reaching EMOC was calculated on NetLogo using the same method as the Spatial Analyst tool/Zonal/Tabulate Area tool in ArcGIS [32]. This method was used to assign residential areas for each class of travel time. Coordinates (x,y) of residential data (LC-Residential) are read and stored through the scanning process:

if ([LC-Residential] of patch-here = 1) [set list-ta lput (patch pxcor pycor) list-ta]

The list of the coordinate patch (x,y) is used to obtain areas according to the classes of travel time. The number of patches for each travel time class was calculated as follows:

set count-blue (count patches with [pcolor = 103])

set count-sky (count patches with [pcolor = 94])

set count-green (count patches with [pcolor = 55])

set count-yellow (count patches with [pcolor = 45])

set count-red (count patches with [pcolor = 14])

The estimated number of people in each of the classes of travel time was presented on a graph. For the cost-benefit analysis, the number of people who travel to EMOC in less than 1 hour was compared with the cost of building new infrastructure.

### Verifying and validating the model

The purpose of verification and validation is to gain credibility for the model with decision makers [33]. They will have confidence in the model if it is shown to be a valid and accurate model of the real-world system which it represents.

We verified this model during its development to determine whether the program was working as we had planned. We also checked that the output was correct with a variety of inputs, thus establishing the accuracy of the model.

After verification, we performed validation. The model’s objective is to model scenarios of infrastructure planning to improve access to EMOCs by decreasing travel time. The validation process comprised three steps. First, we made field trips from residential locations to EMOC locations, calculating actual travel time, to check whether the model’s results were similar to the real travel time. Second, we checked the population result with the population data of Kupang district from the district census data [34]. Third, we introduced the model to the district planning officers and midwives to check whether the model fulfilled its purpose. Following verification and validation, we sought and received the consent of the research participants. First, we asked permission from the Governor of NTT province and from the Headman of Kupang district to conduct this research. Then we conducted a meeting with a *bappeda* (district planning agency) and then with midwives in different locations. At the *bappeda* meeting, we obtained written consent of participants for the research activities, including video recording. Fifteen people attended the *bappeda* meeting. Because of the large number of participants (60) at the midwives’ meeting, and limited time, the meeting leaders asked the researcher to read the consent form and then asked the participants directly to give their consent verbally to the research activities. Acceptance of this process indicated that consent had been given for the research activities. Each participant was not identified.

### Ethical considerations

Ethics approval for the study was obtained from the university’s Human Research Ethics Committee (reference number H14016). There was no likelihood of harm or discomfort to participants in this research project. Participants’ activities were recorded using a video camera. Any possible repercussions from criticising current planning processes were avoided by keeping responses confidential and reporting them in an aggregated way so that individual responses could not be attributed. Written informed consent was obtained from *bappeda* participants and verbal consent from midwives.

## Results and discussion

We developed the scenario modelling tool in response to the poor infrastructure and difficult geographic terrain in Kupang district, which has a limited budget and lacks the human resources needed for making priority planning decisions.

The tool can be accessed at https://doi.org/10.6084/m9.figshare.14298770.

The tool has four advantages for infrastructure planning to improve access to EMOC.

**Fig 7:**
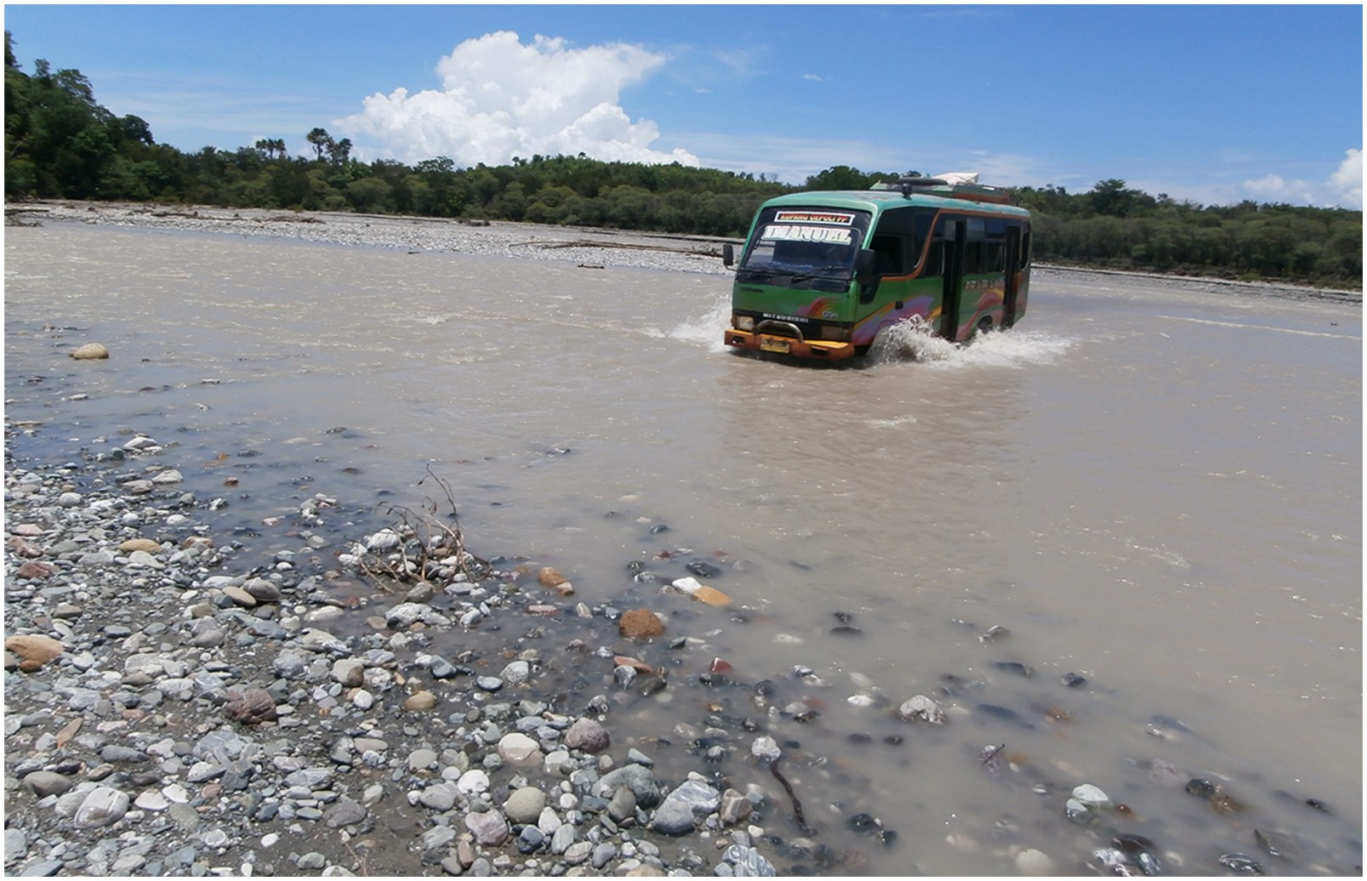
A bus crossing a river in Amfoang.

**Fig 8:**
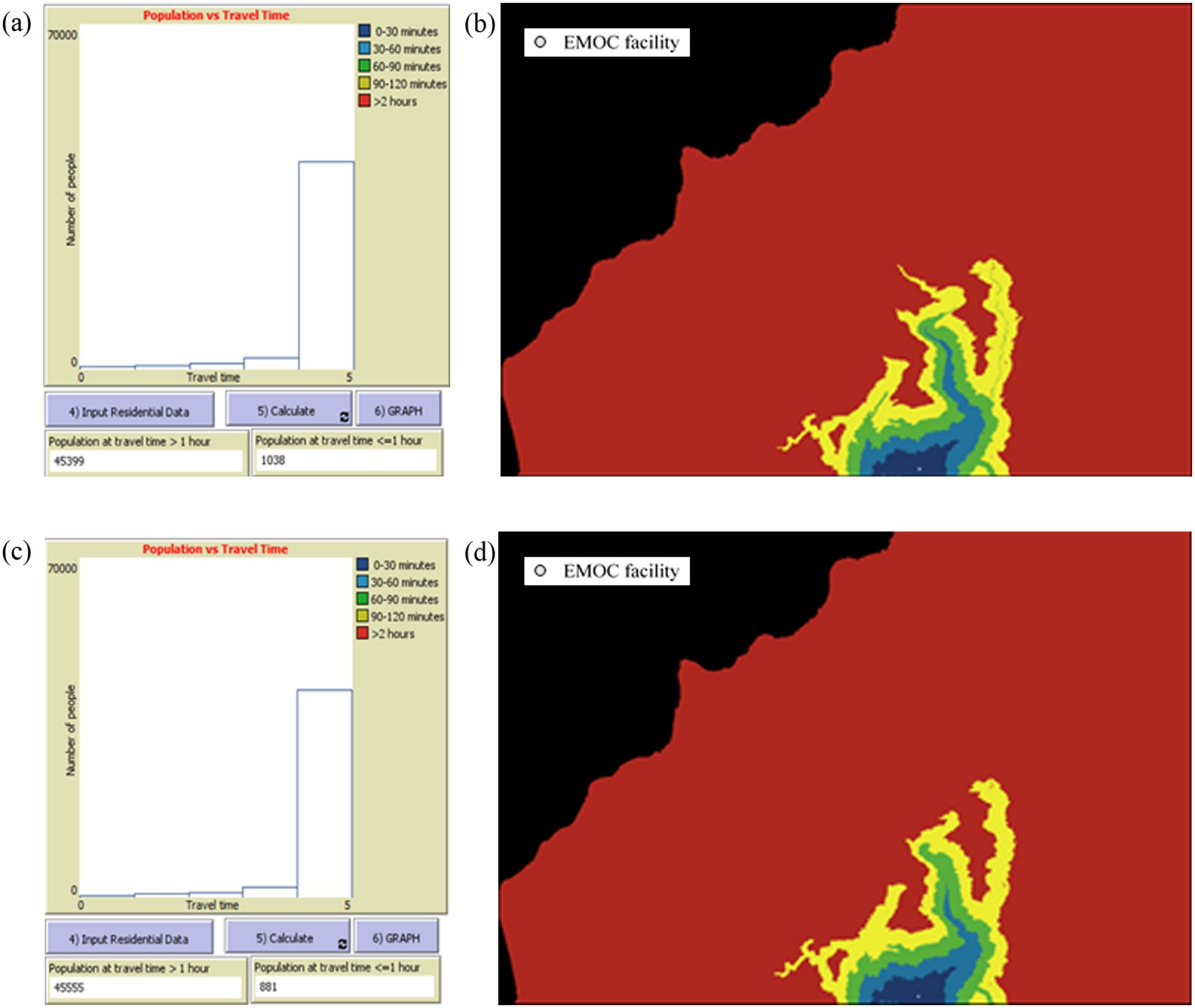
Scenario modelling of EMOC Lelogama in Amfoang. (a), (b) in dry season; (c), (d) in rainy season.

**Fig 9:**
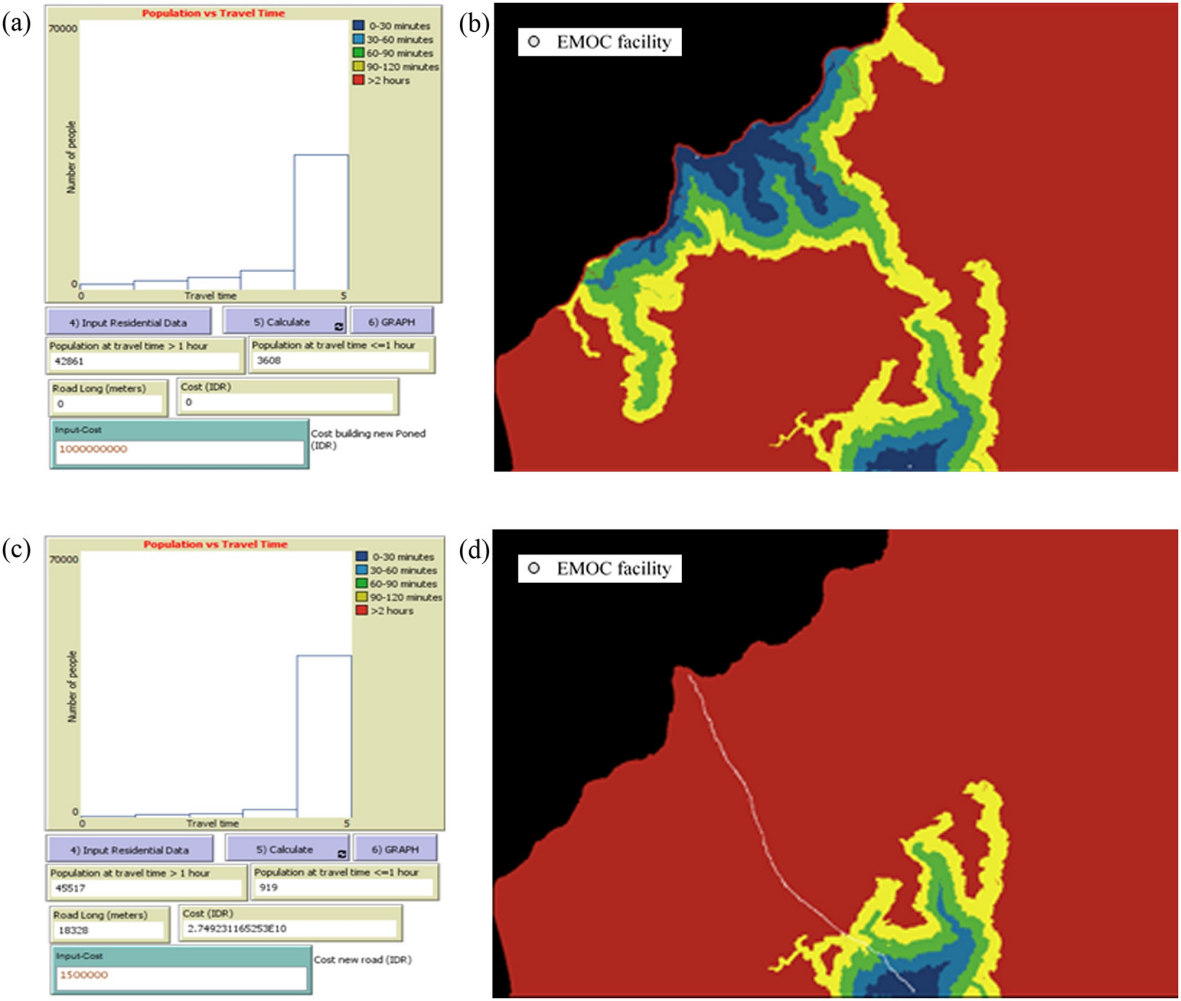
Scenario planning to improve access to EMOC Lelogama in Amfoang. (a) and (b), planning new EMOC; (c) and (d) planning new road.

**Fig 10:**
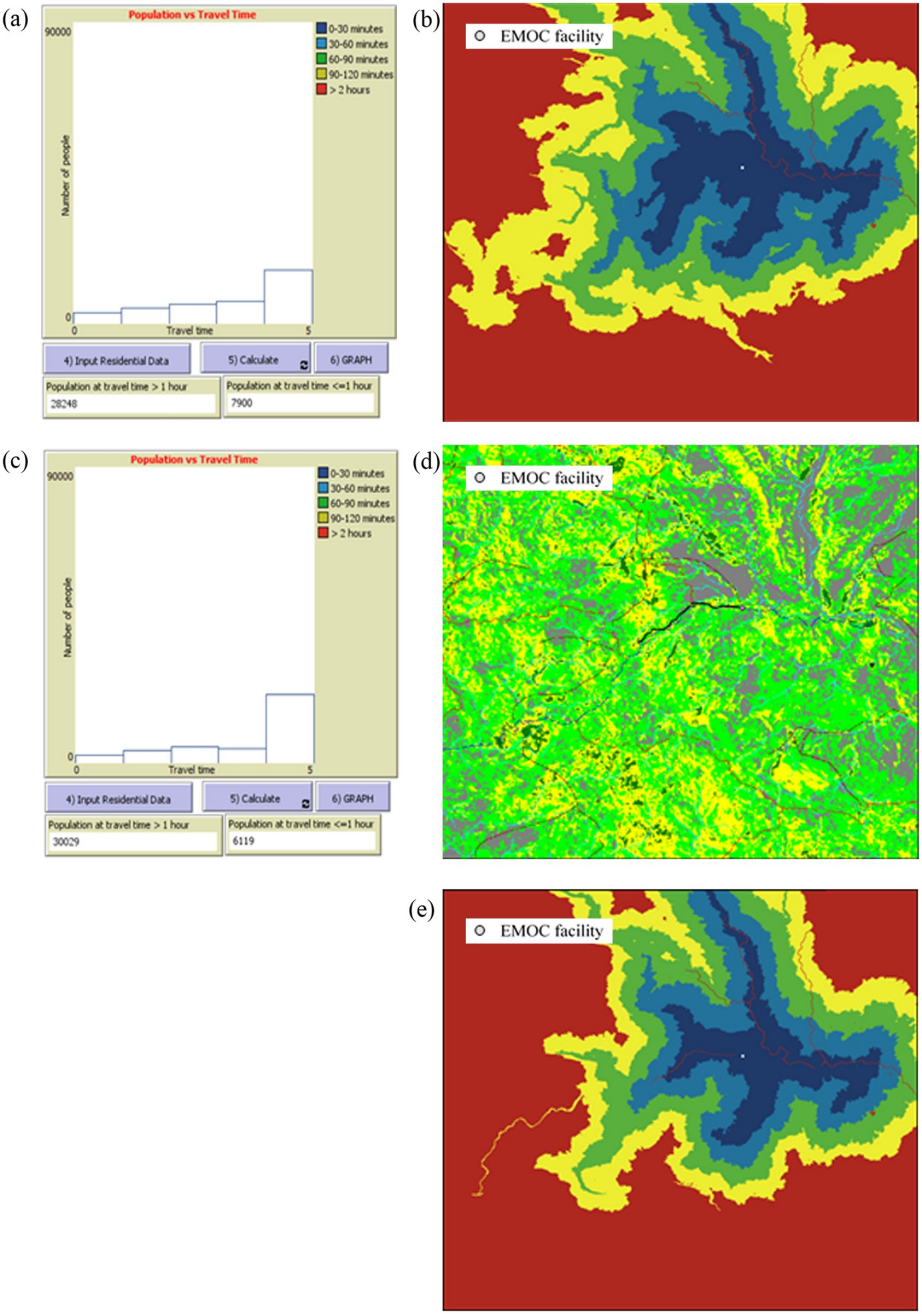
Scenario modelling of EMOC Takari. (a), (b) without barriers; (c), (d), (e) with barriers.

**Fig 11:**
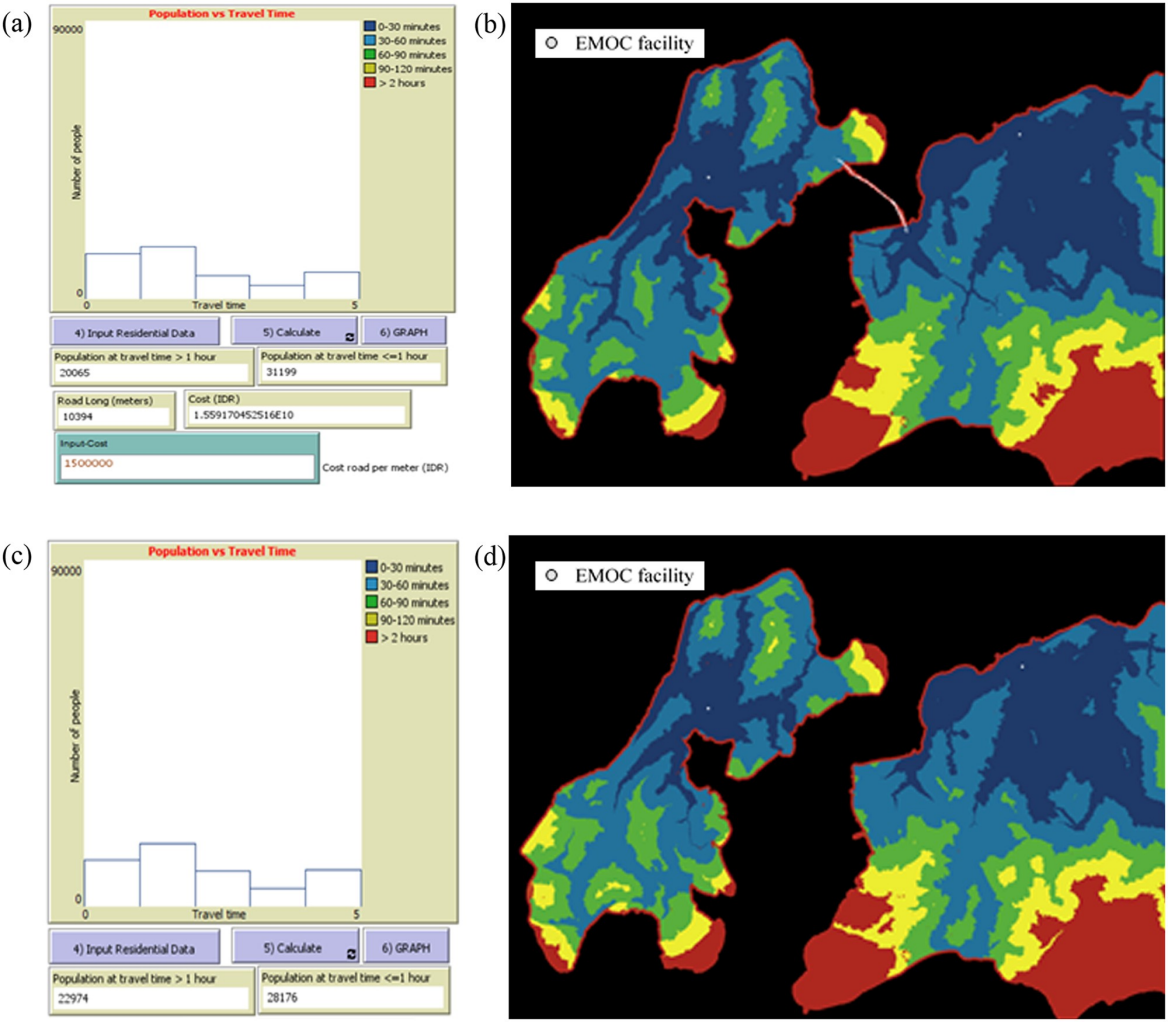
Scenario modelling of PONED Uitao on Semau Island and PONEK RSU on Timor Island. (a), (b) in dry season; (c), (d) in rainy season.

1. **The tool can model accessibility to EMOC based on travel time scenarios in both dry and rainy seasons**. The modelling shows various scenarios of accessibility to EMOC dependent upon weather conditions. As an example, we ran the model to estimate travel times in the dry and rainy seasons for the catchment area of each EMOC, and to know how many people travel to EMOC in different ranges of travel time. According to the Health Ministry of Indonesia (2013) [10], EMOC should be reachable by public transport in 1 hour. The scenario modelling shows that about 80 per cent of the population in Kupang district needed more than 1 hour to reach EMOC in both the rainy and the dry season (Table 2).
  In the rainy season, the number of people who require more than 1 hour to travel to EMOC is slightly higher than in the dry season. For areas with difficult geographic conditions and poor infrastructure such as Amfoang (Fig 7), the travel time model shows that most areas in Amfoang are in the red zone.
  This means that, in most parts of Amfoang, travel time to EMOC is more than 2 hours (Fig 8a–8d). Screen shots of graphs show number of people in each travel time zone in (a) dry season and (c) rainy season. The maps show the travel time model in (b) dry season and (d) rainy season. Media sources confirmed that the communities in six subdistricts in Amfoang (Amfoang Timur, Amfoang Utara, Amfoang Selatan, Amfoang Barat Laut, Amfoang Barat Daya, Amfoang Tengah) were isolated during the rainy season [35].
2. **The tool can be used to compare alternative infrastructure planning scenarios to improve access to EMOC**. For example, in Amfoang, the tool can show which planning proposal is better: building a new EMOC facility or constructing a new road. Building a new facility would increase the number of people who can reach EMOC in less than 1 hour by 3600 (Fig 9a and 9b); constructing 18 km of new road would increase access to EMOC in less than 1 hour for only 900 people (Fig 9c and 9d).
  The cost of constructing the new road is (in Indonesian Rupiah) about IDR27 000 000 000 (USD1 917 000), which is twenty-seven times the cost of building a new EMOC (~ IDR1 000 000 000 or USD71 000). Based on these results, clearly the best plan is building a new EMOC facility: the cost is much less, and it benefits more people than constructing a new road.
3. **The tool enables the users to identify and mark poor infrastructure in the district, taking into account barriers such as floods, damaged roads and broken bridges**. The level of barriers can be determined using the sliders tool on the scenario modelling interface. For example, we created a barrier in the catchment areas of the EMOC in Takari. We assumed that there is a damaged road (shown as a black line in Fig 10d) to the EMOC in Takari. The damaged road caused the number of people taking more than 1 hour to reach EMOC to increase from 28 248 people before the barrier occurred (Fig 10a and 10b) to 30 029 people (Fig 10c–10e). Hence, our scenario modelling helps users to understand the impact of barriers on access to EMOC.
4. **The tool can model cross-ocean travel**. For example, in an emergency situation, a pregnant woman on Semau Island must be referred to PONEK RSU on Timor Island. We created scenarios to model travel time of the pregnant woman to PONEK RSU from the PONED on Semau Island. In the dry season, we assumed that the woman could take a boat from Semau Island to Timor Island. However, in the rainy season, we assumed that the boat cannot travel due to high waves.
  The scenario modelling shows that the number of people reaching EMOC in Uitao or PONEK RSU in the dry season in more than 1 hour (Fig 11a and 11b) is less than the number of people reaching these EMOCs in the rainy season in more than 1 hour (Fig 11c and 11d).

**Table 2:** Number of people who travel to emergency obstetric care for more than 1 hour in the dry and rainy seasons for each catchment area.

Emergency obstetric care facility (PONED) catchment area	Dry season	Rainy season
PONED Lelogama: Amfoang Selatan, Amfoang Barat Laut, Amfoang Tengah, Amfoang Utara, Amfoang Timur	73 625	75 038
PONED Uitao and PONEK RSU: Semau, Semau Selatan and Kupang Barat, Nekamese	20 065	22 974
PONED Oekabiti: Amarasi, Amarasi Barat, Amarasi Selatan, Amarasi Timur	66 459	69 357
PONED Oesao: Kupang Timur, Amabi Oefeto, Amabi Oefeto Timur, Sulamu, Kupang Tengah, Taebenu	111 479	115 190
PONED Takari: Fatuleu, Fatuleu Tengah, Fatuleu Barat, Takari	27 609	28 248
**Total**	299 237	310 807

### Evidence-based planning

To determine if our scenario modelling tool is easy to use as evidence for improving access to EMOC, we introduced it to district planning officers (*bappeda*, *badan perencanaan daerah*) and to the representatives of midwives from all districts in East Nusa Tenggara Province. Our case study was done in Kupang district, but we wanted to introduce the tool to other districts as well. The midwives’ meeting was arranged by the Health Department of Nusa Tenggara Timur Province. We introduced the tool to the midwives and trained them to use it. The midwives, who face problems in helping pregnant women in their areas, engaged with the modelling tool, using their local knowledge for the best results. Now this tool is known in all districts in this province.

We also introduced the scenario modelling to *bappeda*, facilitators of the planning process in the district. However, unlike the midwives, they did not have local knowledge of EMOCs and did not engage with the tool.

In our previous study, we found that in the district planning meetings, called *musrenbang* (***mus****yawarah* ***ren****cana pem****bang****unan*), the planning proposals were submitted from *musrenbangdes* at village level. Midwives attend the *musrenbangdes*. This tool can be used by midwives to submit their suggestions about improving access to EMOC. A midwife in the current study said:

This tool is very good. I want to share to my friends at village. So, when *musrenbangdes*, they can convince the village’s officers about planning to improve access to EMOC [PONED]. Then the village officers can bring this planning evidence to the *musrenbangkab*, so this proposed planning can be accepted.

The midwife realised that she needed evidence to submit in the planning process and that she could use scenario modelling to convince the *musrenbang* participants to prioritise her planning proposal. She realised that she should use the scenario modelling in *musrenbang* at village level because she cannot attend the *musrenbang* at district level. Only village representatives go to the next level of *musrenbang* [36] and, as representatives of *musrenbangdes*, they can take the scenario modelling to the next level of *musrenbang* to be used as evidence for planning. The tool makes midwives aware of the importance of using scenario modelling as evidence in submitting planning proposals at *musrenbang*. Previously, midwives did not know how to submit a proposal to increase access to EMOC.

The midwife’s desire to use this tool as evidence is confirmed by comments from *bappeda*:

It depends on its urgent. If there is debate at *musrenbangcam* (planning meeting at subdistrict), we can use this modelling as one of evidence to be considered in making decision of priority planning. For example, we want to compare scoring 3 and 2 of infrastructure planning which both planning ideas are important. But our budget is limited. So, we can use this modelling to choose which planning can improve access to health service quickly. This method is applicable for us.

Both midwives and *bappeda* wanted to use scenario modelling as evidence in the *musrenbang*. Scenario modelling can be used as planning evidence from *musrenbangdes* (planning at village level) to *musrenbangkab* (planning at district level). *Bappeda* facilitate the *musrenbang* process [37]; they form a team (called *tim pemandu*) to guide the *musrenbang* process. There is usually debate about priorities when making planning decisions; with limited budgets, it is difficult to determine which planning option is the most urgent and deserves priority. To overcome this problem, *bappeda* wanted to use this scenario modelling as the basis of planning and decision making to improve access to health services.

### An interactive tool

Scenario modelling can be used easily–like playing a game. Users can test planning scenarios such as adding new roads, adding new PONED, and drawing barriers. They can compare various scenarios of infrastructure planning to improve access to EMOC. This tool provides basic knowledge of GIS for the layperson–for example, midwives without background knowledge of GIS can easily create travel time maps, read and understand the map, and analyse the results.

### Improving the model

Participants made suggestions to improve the scenario modelling:

1. **The scenario modelling tool should use the location of pregnant women**. A midwife explained:
  This tool is suit for advocation. Because it is not easy to ask building road for Puskesmas (clinic). It takes years. If we are only waiting the road exist, maybe many pregnant women will die. It will be good if adding the location of pregnant women on the model. So, we can focus more detail to the exact women who need help.
  The scenario modelling tool has demonstrated that it can be used to analyse EMOC location. We did not include the location of pregnant women in this scenario modelling, since our model is based on the PONED guidelines of the Indonesian Ministry of Health [10], which decree that a PONED should service 50 000–100 000 people, not only pregnant women. Our modelling tool can also be used as an indicator of the need for emergency obstetric care by WHO [38], which uses total population, not only pregnant women, when determining the need for EMOC in a region. Hence, the tool enables health planners to analyse the location of EMOC based on the guidelines from WHO and the Indonesian Ministry of Health.
2. **The scenario modelling should include small islands in its geographic conditions**. The *bappeda* suggested including not only the main islands but also other small islands. One *bappeda* said:
  We have complicated problem with health access. How the community should get better access to health services. Thanks, *ibu* (called the researcher) to introduce travel time modelling for planning process. I suggested that the modelling should consider geographic conditions because this Timor land consists of many islands with different characteristic.

We have presented scenario modelling showing travel between islands, such as from Timor Island (the main island) to Semau Island. However, we only modelled areas that can be captured by NetLogo, which has limited maximum capacity. The model cannot capture all areas of the Kupang district, which consists of many small islands around the main islands. These small islands do not appear on the NetLogo interface. We used Netlogo because it is a free application.

We welcome any suggestions to improve our model. For the future, we recommend adding the location of women aged 15–39 years to the scenario modelling. Spatial distribution of these “at-risk” women of reproductive age can be analysed [39] to determine the number of these women who may need emergency obstetric care in less than 1 hour. A layer for these women could be aggregated into the travel time layers to calculate the number of at-risk women in each travel time zone. We suggest using district census data to compile a dataset of women of reproductive age [24].

### Action plan for the study result

We have created an interactive scenario modelling tool with example data from the Kupang district as a case study. This tool can be used directly by the midwives and *bappeda* in Kupang district in the *musrenbang*. However, to enable the results of this study to be implemented in other areas, we suggest an action plan. That is first, *bappeda* should create the landcover map (step 1 of the method) and residential map (step 2 of the method) for the district as an input to the tool in Netlogo. Second, *bappeda* can then introduce the scenario modelling tool to the district health department. Third, the health department can organise a meeting with village midwives to introduce this tool to them. *Bappeda* and the health department then provide training to the midwives at this meeting to use this tool. The midwives can then use this tool to propose planning to improve access to emergency obstetric care (EMOC) in the *musrenbang*.

### Conclusions

We developed an interactive scenario modelling tool that can be used by a layperson such as a midwife without prior GIS knowledge to support evidence-based planning for *musrenbang* from the village level in the district in eastern Indonesia. The tool can compare planning scenarios using cost-benefit analyses: the cost is the budget of the proposed infrastructure, and the benefit (in this proof-of-concept instance) is the number of people who can travel to EMOC in less than 1 hour. This tool can model travel time influenced by difficult geographic and terrain conditions based on the travel cost of moving around different types of landcover. It enables the midwives to identify and mark poor infrastructure and model travel across the ocean in the district, taking into account the season and problems such as flooding, damage roads, or broken bridges. Using this tool, the midwives will know how to submit a proposal to increase access to EMOC in district planning. The district planning agency (*bappeda*) will use this tool as the basis of planning and decision making to improve access to health services.

Our study has important implications for maternal health programs to decrease maternal deaths in low-income and lower-middle-income countries by improving access to emergency obstetric care in areas with difficult geographic conditions and poor infrastructure, lack of human resources, and limited budgets. This finding could help policymakers carry out effective planning to improve maternal health care service delivery and contribute to the achievement of Sustainable Development Goal #3 (SDGs), which emphasises the decreasing of the global maternal mortality ratio to less than 70 per 100 000 live births by 2030.
