# Correction: Scenario modelling as planning evidence to improve access to emergency obstetric care in eastern Indonesia

**DOI:** 10.1371/journal.pone.0261392

**Published:** 2021-12-09

**Authors:** Frederika Rambu Ngana, A. A. I. N. Eka Karyawati

In the Development process subsection of the Methods, there is an error in the first equation. Please view the complete, correct equation here:

$$TT=\frac{30}{\left(Travel\;speed\times 1000/3600\right)}$$
